# The Impact of Diagnostic Code Misclassification on Optimizing the Experimental Design of Genetic Association Studies

**DOI:** 10.1155/2017/7653071

**Published:** 2017-10-18

**Authors:** Steven J. Schrodi

**Affiliations:** ^1^Center for Human Genetics, Marshfield Clinic Research Institute, Marshfield, WI, USA; ^2^Computation and Informatics in Biology and Medicine, University of Wisconsin-Madison, Madison, WI, USA

## Abstract

Diagnostic codes within electronic health record systems can vary widely in accuracy. It has been noted that the number of instances of a particular diagnostic code monotonically increases with the accuracy of disease phenotype classification. As a growing number of health system databases become linked with genomic data, it is critically important to understand the effect of this misclassification on the power of genetic association studies. Here, I investigate the impact of this diagnostic code misclassification on the power of genetic association studies with the aim to better inform experimental designs using health informatics data. The trade-off between (i) reduced misclassification rates from utilizing additional instances of a diagnostic code per individual and (ii) the resulting smaller sample size is explored, and general rules are presented to improve experimental designs.

## 1. Introduction

Clearly, a wealth of important clinical information is contained within large electronic health record (EHR) systems. Such information can be an invaluable resource for measuring disease prevalence [1] and disease comorbidity [2], the association between birth month and disease susceptibility [3], the prediction of outcomes [4], the measurement of economic impact of health care [5], and the discovery of etiological factors [6]. A key feature of these data is in the diagnostic codes given by medical professionals to patient records. However, the accuracy of inferring disease phenotypes from electronic diagnostic codes can vary widely across diseases and is often subject to high degrees of error [7–10]. These studies have noted the substantial misclassification effects from the use of electronic diagnostic code data, sufficient to undermine experiments utilizing cases and controls defined by the International Classification of Diseases (ICD) codes alone. The ICD coding system is instituted by the World Health Organization and has been adopted in the United States by the National Center for Health Statistics. More sophisticated approaches to disease classification, such as those using a variety of EHR data and machine learning methods, are difficult to generalize across all diseases and implement in a high-throughput manner. That said, I anticipate that machine learning methods applied to problems of phenotype prediction using EHR variables as features in the predictive modeling will eventually supplant the sole use of ICD code data. Until that time, the use of ICD data may still have utility in initial screens, to be subsequently validated through methods with higher positive and negative predictive values.

## 2. Related Work

In a general setting, the effect of phenotypic misclassification on statistical power of genetic association studies has been previously explored [11–14]. Edwards and colleagues characterized the noncentrality parameter in asymptotic power distributions given the presence of phenotypic misclassification [11]. The authors use cost functions to capture the effect of misclassification and show that the cost of misclassifying a control as a case becomes large and the cost of misclassifying a case individual as a control becomes small as the disease prevalence becomes small. Similarly, Ji et al. also investigated the calculation of a noncentrality parameter capturing phenotype errors for subsequent use in a likelihood ratio test for genetic association studies [12]. Later, Gordon and colleagues showed how to incorporate misclassification error rates into a trend test for genetic association in case/control studies [13]. More recently, Manchia and colleagues investigated the impact of heterogeneity within a clinical phenotype on genetic association [14].

Considering ICD data with misclassification, the type I and type II error rates for genomic association studies were recently thoroughly explored by Duan et al. [15]. The Duan et al. study found little inflation in false-positive rates, but not in considerable false-negative rates under certain allele frequency, effect size, and disease prevalence parameters. In the context of initial screens of ICD codes in EHR systems, several studies have investigated the relationship between the number of instances of particular ICD codes and the measures of diagnostic utility [1, 16–18]. In general, the accuracy of diagnoses improves with the number of instances of the code; however, this is at the expense of smaller sample sizes/increasing false negatives. Hence, there is a trade-off between type I and type II error rates with the number of ICD code instances used to define a disease. In this work, I investigate this trade-off and provide a framework for determining highly powered EHR-based experimental designs using diseases defined by different numbers of instances of ICD codes.

## 3. Materials and Methods

For a large genetic association scan of using ICD data, define a simple disease classification scheme such that cases are those individuals with *x* instances of a particular ICD code. Consider a design where individuals with ambiguous numbers of instances (*i*) of the code (i.e., 0 < *i* < *x*) are excluded from the analysis. Further consider a comparison of well-defined cases (i.e., those with at least *x* instances) against a large, fixed set of controls. With regard to the genetics, restrict the methods to biallelic markers with minor alleles segregating in the population at a frequency of at least 1% single-nucleotide polymorphisms (SNPs). Define the alleles at a SNP contributing to the susceptibility of the disease as *A*_1_ and *A*_2_. Let the relative risk of the minor allele, *A*_2_, be *R*, such that *R* = *P*(*A*_2_ | cases)[*P*(*A*_2_ | controls)]^−1^. Let the frequency of *A*_2_ in the general population be *q*. Accordingly, 1 − *q* is the frequency of *A*_1_. Define *n*_*x*_ as the number of cases obtained from the definition of having at least *x* instances of the ICD code being evaluated. Set the number of controls as *m*, such that *m* ≫ *n*_*x*_. Assume that the *A*_2_ frequency in controls is approximately *q*. Model the decrease in the misclassification proportion within cases as *x* increases with a monotonic function *f*(*x*), such that the expected number of truly positive cases is *n*_*x*_[1 − *f*(*x*)]. The form of *f*(*x*) may vary considerably for different ICD codes. Lastly, let *α* be the statistical threshold for determining a positive finding in analyses where *p* value < *α*. The statistical test of genetic association considered is the binomial test of proportions which evaluates the null hypothesis of no correlation between the frequency of *A*_2_ and the disease status.

Statistical power will be used to evaluate the impact of increasing *x* and the resulting experimental design. Under the model specified above, the power to detect association at an autosomal SNP, 1 − *β*, is calculated by the approximation as follows:

(1) $$\begin{array}{c} \Phi \left[\sqrt{\frac{N{\left(q-s\right)}^{2}}{q\left(1-q\right)+s\left(1-s\right)}}-z_{1-\alpha /2}\right], \end{array}$$

where  *Φ* is the standard Gaussian cumulative distribution function, *z* is the inverse standard Gaussian score, *N* = 4*n*_*x*_*m*/*n*_*x*_ + *m*, and *q* and *s* are the *A*_2_ frequencies in controls and cases, respectively. Using Bayes' theorem, the expected frequency of *A*_2_ within cases under the misclassification model is given by

(2) $$\begin{array}{c} s=f\left(x\right)q+Rq\left[1-f\left(x\right)\right]{\left[1+\left(R-1\right)q\right]}^{-1}. \end{array}$$

To model the decrease in the misclassification rate with increasing numbers of ICD code instances, consider the simple decay function for *f*(*x*):

(3) $$\begin{array}{c} f\left(x\right)={\left(1-\delta \right)}^{x}, \end{array}$$

where *δ* is the parameter that can be estimated for each ICD code. Similarly consider the following form for *n*_*x*_ as a function of *n*_*x*=1_ to model the reduction in the number of cases defined by using increasing numbers of instances of an ICD code:

(4) $$\begin{array}{c} n_{x}=n_{x=1}{\left(1+\varepsilon \right)}^{-x}, \end{array}$$

where *ε* is the parameter that captures the rate of decline in case numbers as the definition for case status becomes more stringent with the use of larger numbers of ICD code instances and can also be estimated for each ICD code. The machinery is now in place for the calculation of statistical power to detect disease association at a genetic marker using data from linked ICD coding systems.

## 4. Results and Discussion

The above model is used to conduct an exploration of the impact of ICD code definitions on power. To obtain a value of *x* which maximizes power to detect genetic association, one can numerically solve the following differential equation for *x*:

(5) $$\begin{array}{c} \frac{\partial }{\partial x}\left[\frac{N{\left(q-s\right)}^{2}}{q\left(1-q\right)+s\left(1-s\right)}\right]=0. \end{array}$$

The solution to (5) can be solved through standard numerical methods applied to solving

(6) $$\begin{array}{c} n_{x=1}\left[{\left(1-\delta \right)}^{2}-1\right]\left(q-1\right){\left(R-1\right)}^{2}\left[F_{1}F_{2}\text{ln}\left(1-\delta \right)+F_{3}\left(F_{4}-F_{5}\right)\text{ln}\left(1+\varepsilon \right)\right]=0, \end{array}$$

where

(7) $$\begin{array}{c} F_{1}=-{\left(1-\delta \right)}^{x}\left[{\left(1+\varepsilon \right)}^{x}m+n_{x=1}\right]\left[q\left(R-1\right)+1\right],\\ F_{2}={\left(1-\delta \right)}^{x}+2q\left(R-1\right)\left[{\left(1-\delta \right)}^{x}+1\right]+R-R{\left(1-\delta \right)}^{x}+3,\\ F_{3}=m{\left(1+\varepsilon \right)}^{x}\left[{\left(1-\delta \right)}^{x}-1\right],\\ F_{4}={\left(1-\delta \right)}^{x}+1+\left[{\left(1-\delta \right)}^{2x}+1\right]q^{2}{\left(R-1\right)}^{2}+R-R{\left(1-\delta \right)}^{x},\\ F_{5}=q\left(R-1\right)\left\{R{\left(1-\delta \right)}^{x}\left[{\left(1-\delta \right)}^{x}-1\right]-{\left(1-\delta \right)}^{x}-{\left(1-\delta \right)}^{2x}-2\right\}. \end{array}$$

**Figure 1:**
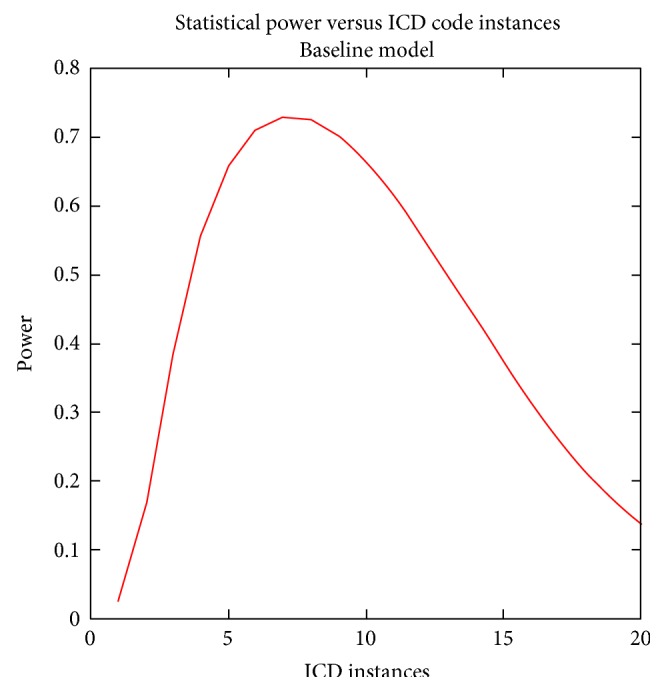
Statistical power versus ICD code instances, baseline model. From the mathematical model specified, power was calculated using the set of parameters from the baseline model. The results show the trade-off between the sample size, misclassification rates, and statistical power to detect genetic association. For the baseline model, the peak of power occurs when the number of instances is 7.

The closest integer value to the value of *x* that solves this continuous equation can be used to optimize the power for a given set of parameters. To exemplify the use of this approach, let *m* = 10,000, *n*_*x*=1_ = 400, *R* = 2, *q* = 0.20, *δ* = 0.15, and *ε* = 0.15. Call this set of parameters the baseline model. *x* = 7.2265 solves the differential equation. Therefore, using seven instances of an ICD code will yield the optimal design weighing the trade-off between the case sample size and the misclassification. For that set of parameters, Figure 1 shows the power curve for this set of parameters.

**Figure 2:**
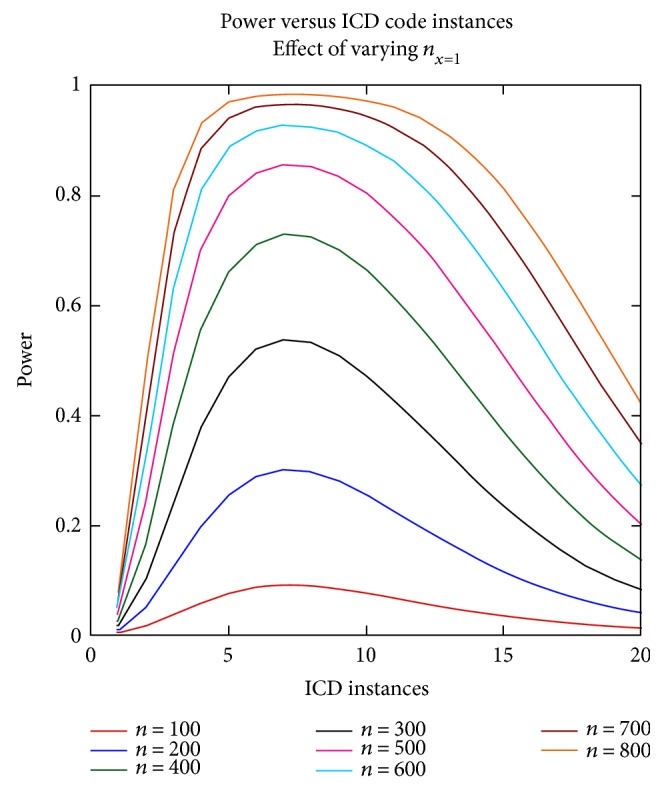
Power versus ICD code instances, effect of varying *n*_*x*=1_. The baseline level was used to generate this figure with the exception of *n*_*x*=1_, which varied from 100 to 800, and the resulting power was calculated for each number of ICD instances.

To investigate the power curves, varying the baseline number of cases (*n*_*x*=1_), the calculations were performed as the *n*_*x*=1_ varied from 100 to 800. Visual inspection shows the peak of power at approximately 7 instances.  Figure 2 shows the results.

**Figure 3:**
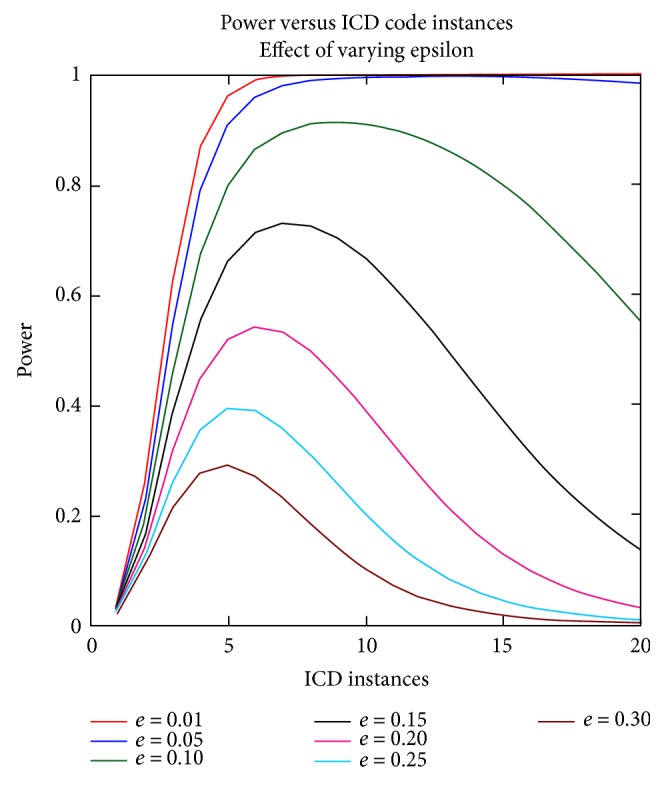
Power versus ICD code instances, effect of varying epsilon. The epsilon parameter varied in the baseline model from 0.01 to 0.30, and the power to detect was subsequently calculated.

**Figure 4:**
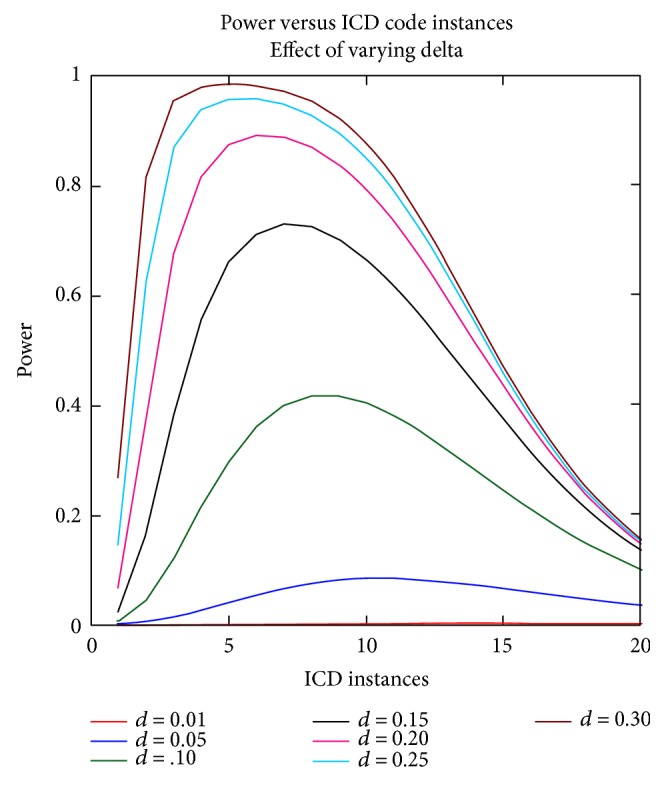
Power versus ICD code instances, effect of varying delta. To explore the effect of the delta parameter on the power calculations, the baseline model was modified to include values of delta from 0.01 to 0.30. The power to detect genetic association was calculated across these delta parameter values.

Next, to determine the role of the *δ* and *ε* parameters on the power curves, the calculations were performed fixing the other parameters. Figures 3 and 4 display these results.

## 5. Conclusions

Genetic data linked to longitudinal electronic health records can serve as a very useful tool in modern disease genetics. However, misclassification present in ICD coding systems can severely hamper large-scale screens using those codes for the purpose of genetic association studies. This work has described a simple approach to better understand the impact of misclassification present in EHR systems for the purpose of optimizing experimental designs that screen numerous ICD codes in genetic association studies. Under the mathematical models considered, the methods offer an approach to select the number of instances of an ICD code for the purpose of defining cases and obtaining an optimal experimental design for the identification of genetic markers. Additional work is needed in this area to improve disease classification schemes for genetic association studies as well as for other investigations.

## References

[1] McCarty C. A., Mukesh B. N., Giampietro P. F., Wilke R. A. (2007). Healthy people 2010 disease prevalence in the Marshfield Clinic Personalized Medicine Research Project cohort: opportunities for public health genomic research. *Personalized Medicine*.

[2] Ko Y., Cho M., Lee J.-S., Kim J. (2016). Identification of disease comorbidity through hidden molecular mechanisms. *Scientific Reports*.

[3] Boland M. R., Shahn Z., Madigan D., Hripcsak G., Tatonetti N. P. (2015). Birth month affects lifetime disease risk: a phenome-wide method. *Journal of the American Medical Informatics Association*.

[4] Glance L. G., Osler T. M., Mukamel D. B., Meredith W., Wagner J., Dick A. W. (2009). TMPM-ICD9: a trauma mortality prediction model based on ICD-9-CM codes. *Annals of Surgery*.

[5] Kinge J. M., Saelensminde K., Dieleman J., Vollset S. E., Norheim O. F. (2017). Economic losses and burden of disease by medical conditions in Norway. *Heath Policy*.

[6] O’Brien S. E., Schrodi S. J., Ye Z., Brilliant M. H., Virani S. S., Brautbar A. (2015). Differential lipid response to statins is associated with variants in the BUD13-APOA5 gene region. *Journal of Cardiovascular Pharmacology*.

[7] Icen M., Crowson C. S., McEvoy M. T., Gabriel S. E., Maradit Kremers H. (2008). Potential misclassification of patients with psoriasis in electronic databases. *Journal of the American Academy of Dermatology*.

[8] Evans J. M., MacDonald T. M. (1997). Misclassification and selection bias in case-control studies using an automated database. *Pharmacoepidemiology and Drug Safety*.

[9] Singh J. A., Holmgren A. R., Noorbaloochi S. (2004). Accuracy of veterans administration databases for a diagnosis of rheumatoid arthritis. *Arthritis and Rheumatism*.

[10] Birman-Deych W., Waterman A. D., Yan Y., Nilasena D. S., Radford M. J., Gage B. F. (2005). Accuracy of ICD-9-CM codes for identifying cardiovascular and stroke risk factors. *Medical Care*.

[11] Edwards B. J., Haynes C., Levenstien M. A., Finch S. J., Gordon D. (2005). Power and sample size calculations in the presence of phenotype errors for case/control genetic association studies. *BMC Genetics*.

[12] Ji F., Yang Y., Haynes C., Finch S. J., Gordon D. (2005). Computing asymptotic power and sample size for case-control genetic association studies in the presence of phenotype and/or genotype misclassification errors. *Statistical Applications in Genetics and Molecular Biology*.

[13] Gordon D., Haynes C., Yang Y., Kramer P. L., Finch S. J. (2007). Linear trend tests for case–control genetic association that incorporate random phenotype and genotype misclassification error. *Genetic Epidemiology*.

[14] Manchia M., Cullis J., Turecki G., Rouleau G. A., Uher R., Alda M. (2013). The impact of phenotypic and genetic heterogeneity on results of genome wide association studies of complex diseases. *PLoS One*.

[15] Duan R., Cao M., Wu Y. (2017). An empirical study for impacts of measurement errors on EHR based association studies. *American Medical Informatics Association Annual Symposium Proceedings*.

[16] Bazarian J. J., Veazie P., Mookerjee S., Lerner E. B. (2006). Accuracy of mild traumatic brain injury case ascertainment using ICD-9 codes. *Academic Emergency Medicine*.

[17] Hebbring S. J., Schrodi S. J., Ye Z., Zhou Z., Page D., Brilliant M. H. (2013). A PheWAS approach in studying *HLA-DRB1∗1501*. *Genes and Immunity*.

[18] Leader J. B., Pendergrass S. A., Verma A. (2015). Contrasting association results between existing PheWAS phenotype definition methods and five validated electronic phenotypes. *American Medical Informatics Association Annual Symposium Proceedings*.

